# Identification of a novel founder variant in *DNAI2* cause primary ciliary dyskinesia in five consanguineous families derived from a single tribe descendant of Arabian Peninsula

**DOI:** 10.3389/fgene.2022.1017280

**Published:** 2022-10-10

**Authors:** Dalal A. Al-Mutairi, Basel H. Alsabah, Bashar A. Alkhaledi, Petra Pennekamp, Heymut Omran

**Affiliations:** ^1^ Department of Pathology, Faculty of Medicine, Health Sciences Center, Kuwait University, Kuwait City, Kuwait; ^2^ Zain Hospital for Ear, Nose and Throat, Kuwait, Kuwait; ^3^ Pediatric Pulmonary Unit, Al-Sabah Hospital, Kuwait, Kuwait; ^4^ Department of General Pediatrics, University Hospital Muenster, Muenster, Germany

**Keywords:** primary ciliary dyskinesia, genetics of ciliopathy, *DNAI2* gene, consanguinity, pulmonary disease

## Abstract

**Introduction:** Primary ciliary dyskinesia (PCD) is caused by dysfunction of motile cilia resulting in insufficient mucociliary clearance of the lungs. The overall aim of this study is to identify disease causing genetic variants for PCD patients in the Kuwaiti population.

**Methods:** A cohort of multiple consanguineous PCD families was identified from Kuwaiti patients and genomic DNA from the family members was analysed for variant screening. Transmission electron microscopy (TEM) and immunofluorescent (IF) analyses were performed on nasal brushings to detect specific structural abnormalities within ciliated cells.

**Results:** All the patients inherited the same founder variant in *DNAI2* and exhibited PCD symptoms. TEM analysis demonstrated lack of outer dynein arms (ODA) in all analysed samples. IF analysis confirmed absence of DNAI1, DNAI2, and DNAH5 from the ciliary axoneme. Whole exome sequencing, autozygosity mapping and segregation analysis confirmed that seven patients carry the same homozygous missense variant (*DNAI2*:c.740G>A; p.Arg247Gln; rs755060592).

**Conclusion:** *DNAI2*:c.740G>A is the founder variant causing PCD in patients belonging to a particular Arabian tribe which practices consanguineous marriages.

## Introduction

Primary ciliary dyskinesia (PCD; OMIM: 244400) is a genetically and clinically heterogeneous group of disorders affecting motile cilia. PCD individuals usually have a history of neonatal respiratory distress and suffer from lifelong symptoms of wet cough, rhinosinusitis and otitis media. Recurrent chest infections eventually lead to bronchiectasis and a progressive decline in pulmonary function (Ratjen et al., 2016; Davis et al., 2019). Nearly one-half of PCD patients display laterality defects, mainly *situs inversus totalis* and other *situs abnormalities*, due to dysmotility of the embryonic motile node monocilia. Male infertility and female subfertility are also associated with PCD (Zariwala et al., 2007; Leigh et al., 2009; Zariwala et al., 2011). The estimated prevalence of PCD worldwide is around 1:16,000, nevertheless it increases to 1:2,265 in the United Kingdom South Asian inbred population (Lucas et al., 2014; O'Callaghan et al., 2010). A similar high incidence is also seen in a highly inbred Kuwaiti population due to practising consanguinity over multiple generations. Early diagnosis is essential for reducing the morbidity of PCD (Shah et al., 2016). The best diagnostic approach includes genetic testing along with determining the ultrastructural defects of the cilia (Shapiro et al., 2018; Dalrymple and Kenia, 2019). Radiological findings of the chest for PCD individuals mainly show atelectasis in the lingua or right middle lobe (Magnin et al., 2012).

PCD is characterized by dysfunction of multiple motile cilia resulting in abnormal mucociliary clearance. Currently, more than 50 PCD genes are identified, mainly encoding for components of the complex structures of axonemes of motile cilia and sperm flagella. The most common PCD gene that is defective in most of PCD individuals of mainly European origin is Dynein Axonemal Heavy Chain 5 (DNAH5) (MIM: 603335), associated with the absence of outer dynein arms (ODA) (Failly et al., 2009). The second most frequent PCD gene associated with the absence of ODA is Dynein Axonemal Intermediate Chain 1 (DNAI1) (Zariwala et al., 2006; Lucas et al., 2014). In general, there are six dynein structural genes associated with PCD and ODA defects. Three genes encode for axonemal ODA heavy chains *DNAH5* (Olbrich et al., 2002; Hornef et al., 2006; Failly et al., 2009), *DNAH9* (Loges et al., 2018), and *DNAH11* (Schwabe et al., 2008; Pifferi et al., 2010; Knowles et al., 2012). Two genes encode for axonemal ODA intermediate chains *DNAI1* (Zariwala et al., 2006) and *DNAI2* (Loges et al., 2008). One gene encodes for axonemal ODA light chains *DNAL1* (Mazor et al., 2011).

The evolutionarily conserved role for *DNAI2* in the assembly of ODA was previously studied in *Chlamydomonas* *oda6* mutant strain; pathogenic variants in *IC2* (*IC69*) orthologous to *DNAI2* lead to disrupted ODA complexes (Fowkes and Mitchell, 1998). Another study shown pathogenic variants in the *C* *hlamydomonas* ortholog (*IC69*) caused reduction in motility of mutant strain (*oda6*) compared to the wild type parent and loss of ODA (Pennarun et al., 2000). The same phenotype was also seen in the *pf28* (*oda2*) mutant strain harbouring pathogenic variants in the γ-dynein heavy chain orthologous to *DNAH5*, indicating the role of the DNAI2 and *DNAH5* orthologs in the assembly of ODA in both human and *Chlamydomonas reinhardtii* (Mitchell and Rosenbaum, 1985).

Dynein Axonemal Intermediate Chain 2 (DNAI2) located in human on chromosome 17q25 (MIM no. 605483) is a component of the ODA complex and is essential for the assembly of this multimeric complex. *DNAI2* was initially cloned and characterized as a PCD gene using the candidate gene approach (Loges et al., 2008). Pathogenic variants in *DNAI2* are a rare cause of PCD. Early studies did not show disease-causing variants of this gene in 16 PCD families, analysed with microsatellite marker alleles concordant for loci on chromosome 17q25 (*DNAI2* locus) (Pennarun et al., 2000; Loges et al., 2008). Homozygosity mapping performed in other studies identified linkage to the *DNAI2* locus and a homozygous splice variant (*DNAI2*:IVS11+1G<A) in all affected individuals of a Jewish Iranian family (Loges et al., 2008). Moreover, in the same study sequence analysis of additional 105 unrelated patients (48 presenting with ODA defects) identified a homozygous nonsense variant (*DNAI2*:c.787C>T; R263X) in a German PCD individual and a homozygous splice variant (*DNAI2*:IVS3-3T<G) in a Hungarian PCD family (Pennarun et al., 2000). Overall, *DNAI2* variants were found in ∼2% of all PCD families and 4% of PCD families with documented ODA defects (Loges et al., 2008). In this study, a founder variant in *DNAI2* was identified in seven patients belonging to the same Arabian tribe.

## Methods

### Human subjects

Ethical approval for this project was obtained from Ministry of Health Research Ethics Committee (Ethics ID: 62/2013). This study was carried out with the permission of all participating family members; informed written consent was obtained from adult participants and from the parents of children for collection of blood samples from the patients and non-affected parents as well as the nasal biopsy. Pedigrees for the families were constructed using Cyrillic version 2.1 (http://www.cyrillicsoftware.com/) according to the Supplier’s guidelines. Radiological data were collected for most of the patients under study to correlate the genotype with the clinical phenotype.

### Genomic DNA and exome sequencing

**FIGURE 1:**
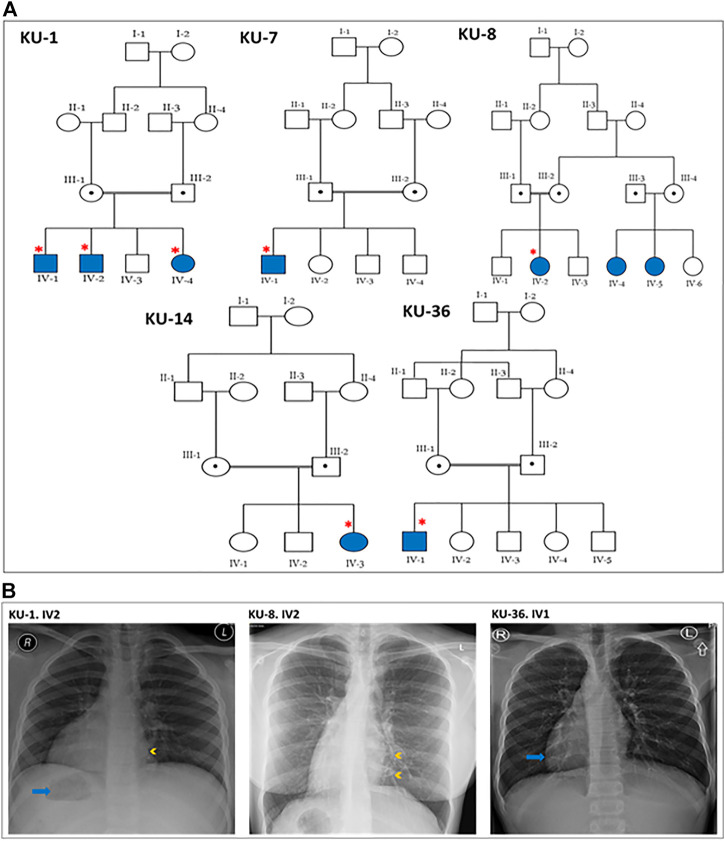
Pedigrees and the chest X-rays for the PCD individuals. **(A)** shows the five pedigrees of families under study. The DNA samples and nasal biopsies were taken from the affected individuals (highlighted with asterisks). For KU-1.IV2 patient, only DNA samples were taken later for Sanger sequencing. **(B)** shows selected chest X-rays for three of the patients. The three patients have *dextrocardia* and a right-sided gastric shadow (arrow), in keeping with *situs inversus*. Patient KU-1.IV2 has a small patch of consolidation seen in the paracardiac region of the left lung (arrowhead). Patient KU-8.IV2 has diffuse bronchiectasis as seen in the medial left lower zone (arrowheads). Patient KU-36.IV1 shows dextrocardia (arrow) consistent with *situs inversus*.

Genomic DNA was extracted from whole blood using the QIAamp mini-isolation kit (Qiagen); concentrations were determined by UV spectrophotometry using a Nanodrop N1000 (Nanodrop Technologies Inc.). Exome sequencing of genomic DNA was performed for six individuals highlighted with asterisks (Figure 1) by the Cologne Centre for Genomics, Germany. Target enrichment was performed, following manufacturer’s protocols, using SureSelect hybridization capture reagents with V6 (Agilent Technologies). Enriched library preparations were sequenced on HiSeq 2500 platform (Illumina). Linkage analysis using exome data was performed using pipeline-produced variant call format (VCF) files.

### Autozygosity mapping and variant screening

Genetic screening using autozygosity mapping was performed using software written at the University of Leeds, United Kingdom (Carr et al., 2009; Carr et al., 2011a; Carr et al., 2011b; Carr et al., 2013; Watson et al., 2015). Initially, AgileMultiIdeogram software was used to visualize the homozygous intervals using exome data for linkage in which all the homozygous intervals are displayed against a circular ideogram for the 22-autosomal chromosomes for the relative patients (Carr et al., 2013). The reference of genome annotation used was Human Genome Build hg19 (UCSC genome browser).

Primers for variant confirmation and segregation analyses were designed using Primer3 software (http://frodo.wi.mit.edu/primer3/). The exon sequences were obtained from the University of California, Santa Cruz Genome Browser (http://genome.ucsc.edu/) for exon 7 of *DNAI2* gene. For the founder variant rs755060592, the sequence of the forward primer used was “GAT​TTG​AAC​CAA​GCC​CTG​AT” and the sequence of the reverse primer used “GCC​AAC​ATA​GTG​AAG​CAC​CA”.

All targets were amplified using a commercial PCR master mix (Promega, Southampton, United Kingdom). PCR was performed in a total reaction volume of 20 µl. The master mix contained 4 µl of 5x Flexi buffer, 1.2 µl of 25 mM MgCl_2_, 0.4 µl of 10 mM dNTP that contained a mixture of dATP, dCTP, dGTP, and dTTP nucleotides and 1.2 units of TaqFlexi DNA polymerase. The amount of DNA amplified using that master mix was 40 ng of genomic DNA. For all amplicons initial denaturation was at 95°C for 30 s, followed by 30 cycles of 95°C for 15 s, 58°C for 15 s, 72°C for 30 s and a final 300 s extension at 72°C. Amplicons were purified by using ExoSAP-IT exonuclease (USB Corporation, Cleveland, United States) according to the manufacturer’s instructions. PCR products were stained by ethidium bromide and analysed by separation on a 1.5% agarose gel. Sequencing was performed using the BigDye 3.1 kit (Applied Biosystems, Foster City, United States). Sequencing reactions were ethanol-precipitated and re-suspended in HiDi formamide (Applied Biosystems) before analysis on a 3130xl genetic analyzer with a 36 cm capillary array. The Sanger sequencing results were analysed using a GeneScreen software (Carr et al., 2011b)**.**

### Immunofluorescent analyses of nasal biopsies

Respiratory epithelial cells were obtained by nasal brush biopsy and suspended in cell culture medium. Samples were spread onto glass slides, air dried and stored at −80°C until use. The respiratory epithelial cells from healthy control and six PCD-affected individuals highlighted with asterisks (Figure 1) were dual labelled for IF analysis with antibodies against axonemal components (visualized with red fluorescent secondary antibody) and antibodies to acetylated anti-Tubulin (visualized with green fluorescent secondary antibody) as ciliary marker. Nuclei were stained with Hoechst 33342 (blue fluorescent) (Wallmeier et al., 2014). Immunofluorescence images were taken with a Zeiss LSM 800 confocal microscope and processed with ZEN and ImageJ software.

The panel of primary antibodies used for initial screening included mouse monoclonal anti-tubulin (acetylated) antibody (Sigma Aldrich, T6793) to stain ciliary microtubules and rabbit polyclonal antibodies as follows: anti-DNAI2 (1:300) (HPA050565, Atlas Antibodies), anti-DNAH5 (1:300) (HPA037470, Atlas Antibodies), anti-DNAI1 (1:300) (HPA021649, Atlas Antibodies). The secondary antibodies used were highly cross absorbed secondary antibodies, including Alexa Fluor 488-conjugated goat antibodies to mouse (1:1000) (A11029, Molecular Probes, Invitrogen) and Alexa Fluor 546-conjugated goat antibodies to rabbit (1:1000) (A11035, Molecular Probes, Invitrogen).

### Transmission electron microscopy analysis of nasal biopsies

For transmission electron microscopy (TEM), nasal biopsies were taken from the middle turbinate. The ciliated cells were fixed with glutaraldehyde (2.5%) in Sorensen’s phosphate buffered (pH 7.4). After post-fixation, the samples were treated with osmium tetroxide (1.3%), dehydrated through graded ethanol series and immersed in hexamethyldisilazane, a chemical drying reagent. The samples were embedded in 1,2-Epoxypropan-Epon- mixture (1:1) at 4°C overnight. After polymerisation, several sections were picked out onto copper grids. The sections were stained with Reynold’s lead citrate. TEM was performed with the Philips CM10 (Wallmeier et al., 2014).

### Validating the *DNAI2* founder variant in Arab population

The allele frequency of the identified variant was validated using real time PCR by performing the allelic discrimination test using the same primers designed for Sanger sequencing as described above (Applied Biosystems). Briefly, PCR reactions were carried out using 25 µl reaction mixture containing 25 ng DNA, 1X Taqman primer/probe mix (Applied Biosystems), 1X genotyping Taqman master mix (Applied Biosystems) and sterile DNA/RNA free water. In each experiment, positive (cases for which genotype was confirmed by Sanger sequencing) and negative (water) controls were included. A batch of an ethnically matched Arab control DNA panel collected from 100 normal subjects that belong to different Arabian tribes was run in parallel. Genotyping steps were performed in a 7500 Fast Real-Time PCR System according to the manufacturer’s instruction (Applied Biosystems).

## Results

### Autozygosity mapping reveals a founder homozygous variant in *DNAI2*

Patients were referred for genetic assessment to clinics run by the Ministry of Health in Kuwait. Most patients were suspected to carry disease causing hereditary variants as more than one individual in their families exhibited the same phenotype. Five unrelated families were independently ascertained with PCD diagnosis in a cohort of 50 multiplex families recruited from different hospitals in Kuwait; affected family members showed clinical features typical of the hallmark disease of PCD (MIM: PS244400), mainly neonatal respiratory distress, chronic respiratory disease with symptoms of chronic airway infections, rhinosinusitis, otitis media, bronchiectasis, and *situs inversus totalis*. The ethnic background of all the patients under this study are Asian Arabs belonging to the same tribe from Arabian Peninsula.

This report summarizes the overall genetic analyses for these five families KU-1, KU-7, KU-8, KU-14, and KU-36 with a total number of seven PCD individuals, all members of the same tribe, which is the largest tribe in Kuwait. As seen in pedigrees (Figure 1A), there are two multiplex families that have more than one affected individual. In addition, the informed consent and the questionnaire indicated that there are many other potential PCD individuals belonging to the same tribe that could not be included on the pedigrees as we faced difficulty in reaching them since they live in isolated areas in the more remote regions of Kuwait.

All seven patients were diagnosed with PCD *via* both genetic studies and structural analysis of the respiratory cilia. The paediatric pulmonologist performed a differential diagnosis for six of the patients and excluded other conditions including cystic fibrosis, asthma, anatomic anomalies and immunodeficiencies. The median age at diagnoses was 2 years, with range 1–12 years. All patients included in this study started showing symptoms during early infancy. The patients were hospitalized during infancy for mild respiratory distress and required oxygen administration. All of the patients reported the following symptoms: wheeze, dyspnea, recurrent pneumonias, a chronic productive cough, and constant nasal mucopurulent secretions. These symptoms gradually became more progressive with age. All individuals participating in our study were asked to undergo serial spirometries in the period following up. Three individuals showed only an obstructive pattern; the other four individuals showed a decline in the respiratory function with both an obstructive and restrictive (mixed) pattern. Infertility was reported in the adult PCD male KU-36. IV-1, but no sperm analysis was available. The most commonly reported specific presentations of PCD was *situs inversus totalis*, which was found in all patients in this study. Previous studies have shown truncated variants in *DNAI2* result in randomization of left/right body asymmetry and develop *situs inversus totalis* due to altered motility of nodal cilia (Nonaka et al., 1998; Loges et al., 2008).

It is important when managing these patients to have a considerate approach. This includes administrating intermittent antibiotic therapy for patients presenting with acute chest exacerbations, and preventive antibiotic therapy (macrolides) for the patients who presented with recurrent chest exacerbations. Chest physiotherapy is also strongly advised, and annual vaccinations against influenza and pneumococcus. None of the patients required surgical intervention for bronchiectasis. According to their radiographic images, three cases underwent high-resolution computed tomography (HRCT). They showed clear evidence of bronchiectasis, including thickened bronchial wall with mucoid impaction (data not shown). The chest X-ray images for three PCD individuals confirm a clinical diagnosis of PCD including *situs inversus totalis,* consolidation and bronchiectasis (Figure 1B).

**FIGURE 2:**
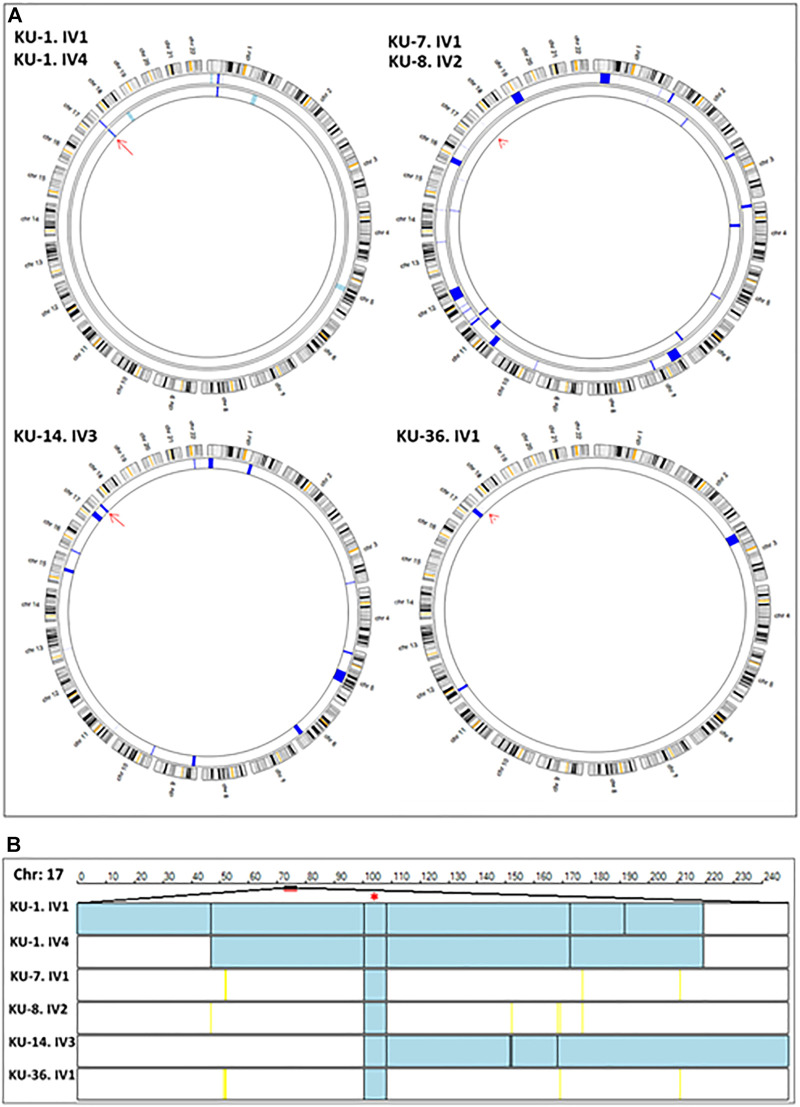
Linkage analyses using exome data. **(A)** summarizes the linkage analysis using AgileMultiIdeogram software and presented in such a way that each ideogram shows the overall linkage for the patients belonging to the same family and each lane accounts for one patient. The density of IBD is represented by the bulk of navy-blue bars for the shared regions of homozygosity (autozygous intervals). The light blue bars specify the unshared ROH regions in multiplex families. In singleton families, having one affected individual, all the detected ROH are displayed as navy blue bars, since there is no shared ROH to be prioritized by the software as seen for families KU-14 and KU-36. Linkage results show the two affected siblings belonging to the KU-1 family have an exclusive autozygous interval across the *DNAI2* locus highlighted by a red arrow. Linkage analysis was performed for the two patients from families KU-7 and KU-8 together since they are distantly relatives and the results show a minute IBD interval across the gene locus that indicated by the red arrowhead. The same results were seen for KU-36, although this patient has another significant ROH interval upstream of the *DNAI2* locus suggesting that the founder variant in *DNAI2* has a very high penetrance. **(B)** shows the linkage scan for the six patients inheriting a founder variant in *DNAI2* using AgileVCFMapper software. Linkage scan highlighted a unique autozygous interval indicated by two black lines at chromosome 17 across the *DNAI2* locus that emphasised by red asterisk. In this figure, each row represents the genotypes for chromosome 17 for one patient as illustrated in the figure. In mapped autozygous regions, the blue bars indicate the extent of autozygous regions. The black vertical lines represent concordant “common homozygous variants” that are shared among patients with the same founder variant. The yellow vertical lines indicate the positions of nonconcordant “rare homozygous variants”. White bars indicate the “no-calls” and that predominantly seen in autozygosity mapping using exome data.

Linkage analysis using autozygosity mapping approach has been performed for all the available affected individuals belonging to the same family, such as family KU-1 in one setting together. This was also performed for the two patients belonging to families KU-7 and KU-8 since the mothers of the affected children are distant relatives. Linkage analysis was performed for families KU-14 and KU-36 separately. The linkage analysis was performed for all the subjects using the lowest level of allele read frequency 10% instead of 25%, which is the default setting for obtaining VCF format for linkage using the exome data. Decreasing the allele frequency was done for the purpose of increasing the resolution of linkage especially for the cases that had a minimal shared interval of Identical by Descent (IBD) across the disease gene locus, as typically seen in families KU-7, KU-8, and KU-36 (Figure 2A). In addition, the resolution of linkage analysis using exome data was further increased by using higher capture-V6 that covers bigger regions of non-coding sequence (61 Mb) compared with the standard capture V7 (37 Mb).

The IBD intervals were determined for each patient at the beginning of the study to estimate the shared IBD intervals, especially for either closely or distantly affected relatives. Mapping the shared IBD intervals is the key for disease-gene identification in patients belonging to multiplex families, or in a group of patients belonging to singleton families derived from the same tribe, as presented in this study. The thickness of the IBD interval represents the extent of the autozygous regions (Carr et al., 2012; Carr et al., 2013). The estimated homozygous intervals in each chromosome were precisely determined using AgileVCFMapper software (Watson et al., 2015).

After that, multiple linkage analysis was performed for six PCD individuals using AgileVCFMapper software, which is an Autozygous Variant Viewer application that analyse each chromosome separately (Watson et al., 2015). This analysis enables mapping of the haplotype for shared regions of autozygosity. This was used successfully to determine the autozygous intervals that harbour founder variants shared across several families by estimating the overlapping homozygous and concordant variants shared among unrelated affected individuals, as seen in Figure 2B.

Screening for deleterious variants in the known PCD genes, in a total of 50 PCD genes to date, was performed in parallel with filtering of the variants at the IBD segments that were determined for all affected individuals in the cohort by estimating all regions of homozygosity (ROH); shared ROH was also estimated between the affected siblings for each family (data not shown).

Initial linkage analyses showed that patients belonging to families KU-1, KU-14 and KU-36 have a detectable ROH segment across the *DNAI2* locus at Chr:17 (Figure 2A). Multiple linkage analysis results showed a unique autozygous interval (blue bar) at chromosome 17 across the *DNAI2* locus shared among the six PCD individuals (Figure 2B). Interestingly, this unique autozygous interval can only be detected using a high resolution of linkage SNP-genotyping. As seen in our data, a shared IBD interval that cannot be clearly visualized with AgileMultiIdeogram software in three patients in Figure 2A (KU-7, KU-8, and KU-36) was detected using AgileVCFMapper software (Figure 2B). Multiple linkage results suggested that a pathogenic variant in *DNAI2* is likely disease causing in these families under study. After that, analysis of exome data at the shared IBD interval revealed a homozygous variant (c.740G>A; p.Arg247Gln) at *DNAI2* locus inherited in the six PCD individuals. Sequencing chromatograms for all seven PCD individuals confirm that all the patients are harbouring the same homozygous missense variant detected in *DNAI2.*

The variant was checked using different algorithms and statistical measurements that predict the pathogenicity of the variants. We used VarSome in initial *in silico* analysis to predict pathogenicity of the *DNAI2*:c.740G>A variant. Here, six metatools that each determine a pathogenicity based on the combined evidence from multiple other *in silico* predictors predict this variant to be damaging (MetaLR, MetaSVM, MetaRNN, BayesDel noAF, BayesDel addAF) or pathogenic (REVEL), 16 out of 18 individual prediction programs that largely use dbNSFP version 4.2 database developed for functional prediction and annotation of all potential non-synonymous single-nucleotide variants (nsSNVs) in the human genome predict the *DNAI2*:c.740G>A variant as pathogenic/damaging/deleterious and/or disease causing, including widely used prediction programs such as: DANN, MutPred, Mutation assessor, MutationTaster, Provean or Polyphen-2 (Loges et al., 2008; Mazor et al., 2011; Knowles et al., 2012).

The results of other algorithms are described in more detail: Firstly, Rejected Substitutions (RS), defined as the number of substitutions expected under a neutral model minus the number “observed” (estimated) for a particular alignment (Pazour et al., 2006). Secondly, Polymorphism Phenotyping (PolyPhen), a tool that predicts possible impact of an amino acid substitution on the structure and function of a human protein (Adzhubei et al., 2013). Thirdly, Residual Variation Intolerance Score (RVIS), a gene score based module intended to help in the interpretation of human sequence data (Petrovski et al., 2013). The missense variant in *DNAI2* (c.740G>A) resulting in an amino acid exchange (p.Arg247Gln) has RS = 5.2, PPH = 3 and RVIS = 0.01, all of which indicate that the variant is highly damaging.

Additional *in silico* analyses on protein level showed that this variant is located between WD40 domains 2 (aa208-246) and 3 (aa253-294), essentially right at the end of WD40 2 (UniProt). However, no specific motifs were found in that area by using EMBL-ELM (eukaryotic linear motif) that could help to explain the impact of this variant on DNAI2 protein function.

In the initial stage of the investigation this variant was mapped in Families KU-1, KU-7 and KU-8 and was not validated on Ensembl database. Recently, it has been given a RefSNPs (rs) number (rs755060592). Remarkably, the allele frequency of the normal allele (G) is 100% in all ethnic backgrounds according to the Ensembl genome browser. In this study, it has very high penetrance as it was found to be inherited in patients with low ROH score that belong to families KU-7, KU-8, and KU-36.

**FIGURE 3:**
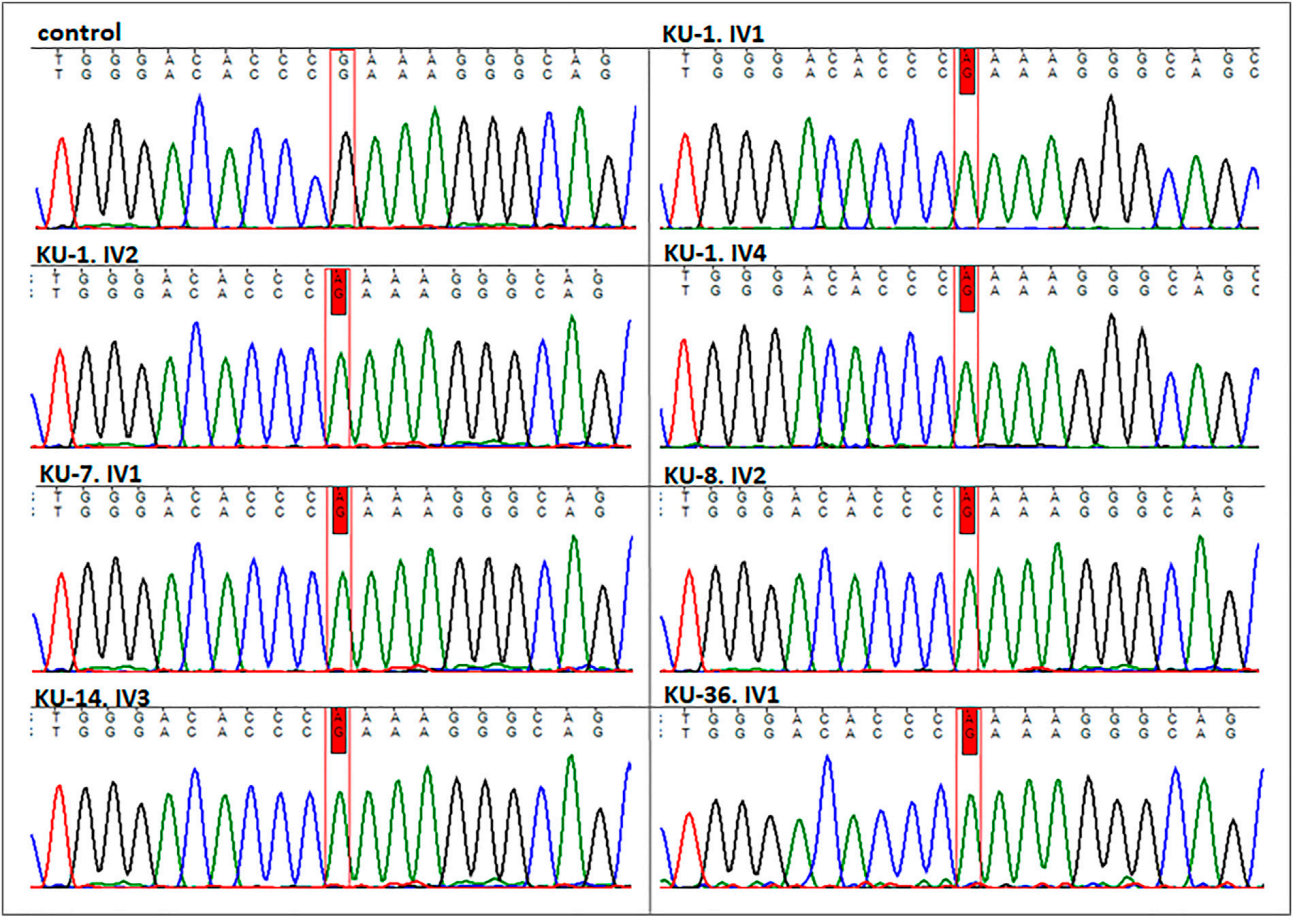
Sequencing chromatographs for the seven PCD individuals. All the patients having homozygous missense variant (*DNAI2*:c.740 G>A; p.Arg247Gln) rs755060592 in exon 7 as illustrated *vs.* heathy individual as control.

**FIGURE 4:**
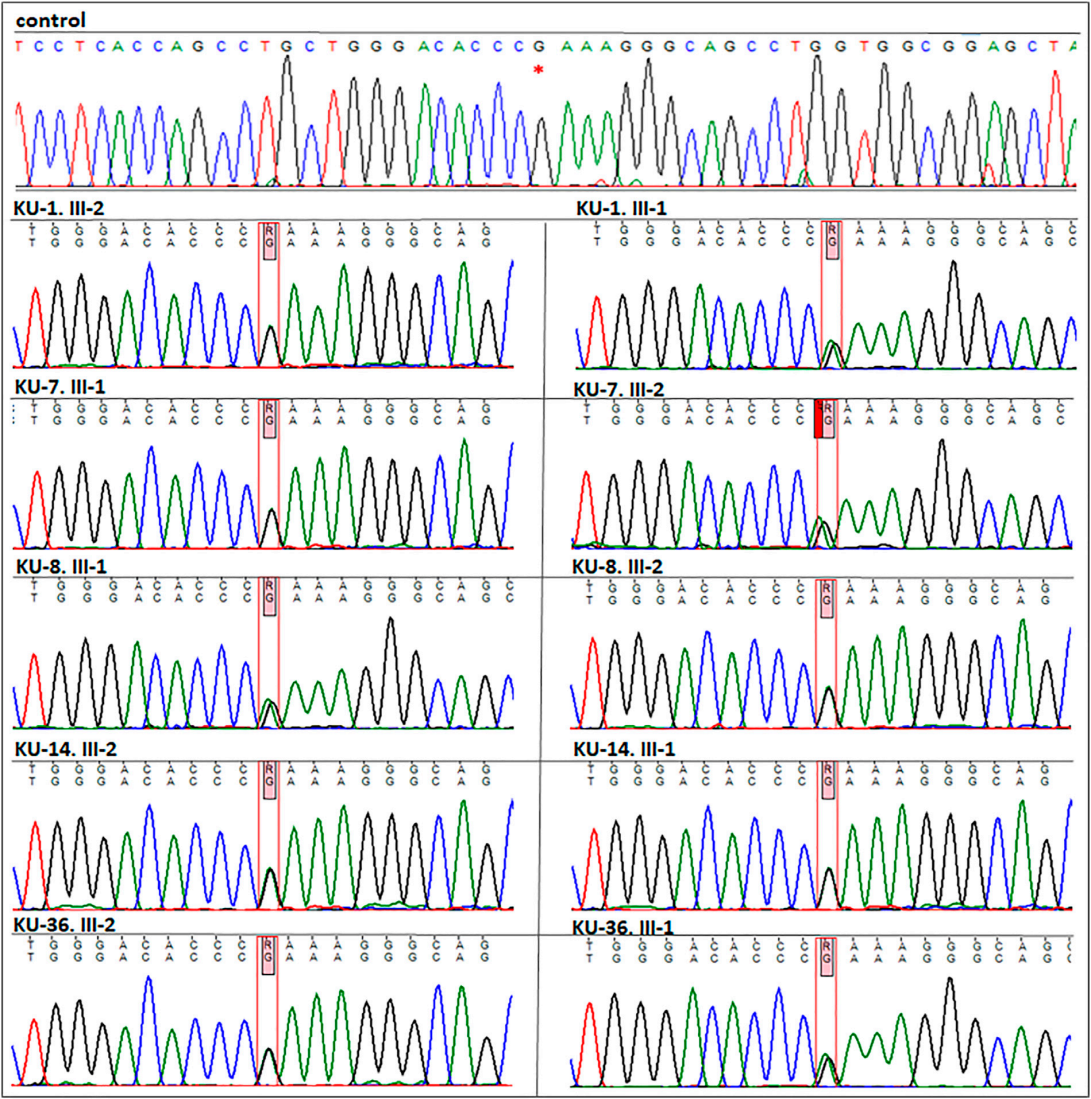
Segregation analysis for the parents of the five families. As seen in this figure the chromatographs for the sequencing results for the five parents, which demonstrate that all the parents are carriers for the missense variant (*DNAI2*:c.740 G>A; p.Arg247Gln) rs755060592 in exon 7. This confirms that the variant is segregated along with the disease phenotype in the seven PCD individuals.

This indicates that the missense variant in *DNAI2* is likely a founder variant causing PCD this tribe (Figure 3). Following the linkage and sequencing analyses, segregation analysis was performed to confirm that the identified variant in seven patients segregates with the disease phenotype. The results of segregation analysis confirmed that all the parents are heterozygous carriers for the founder variant, which indicates that the variant segregates with the disease phenotype and is the pathogenic PCD variant, as seen in Figure 4.

After that, allelic discrimination assay for the *DNAI2* founder variant was performed using RT-PCR. It was performed in order to estimate the carrier rate frequency of the founder variant (*DNAI2*:c.740G>A) using an Arab control panel composed of 100 DNA samples for normal individuals belonging to different Arabian tribes. As shown in Supplementary Figure S3, the genotype for the six PCD individuals is homozygous for the missense variant (A), and all their parents are heterozygous carriers for the wild type allele (G) and the defective allele (A) which is consistent with Sanger sequencing data. Interestingly, the genotypes for the Arab control DNA panel show that all the healthy individuals have the two wild type allele (G). This indicates that the variant is very rare and the carriers are rarely existing in Arab populations.

### Detection of ultrastructural defects of the cilia

**FIGURE 5:**
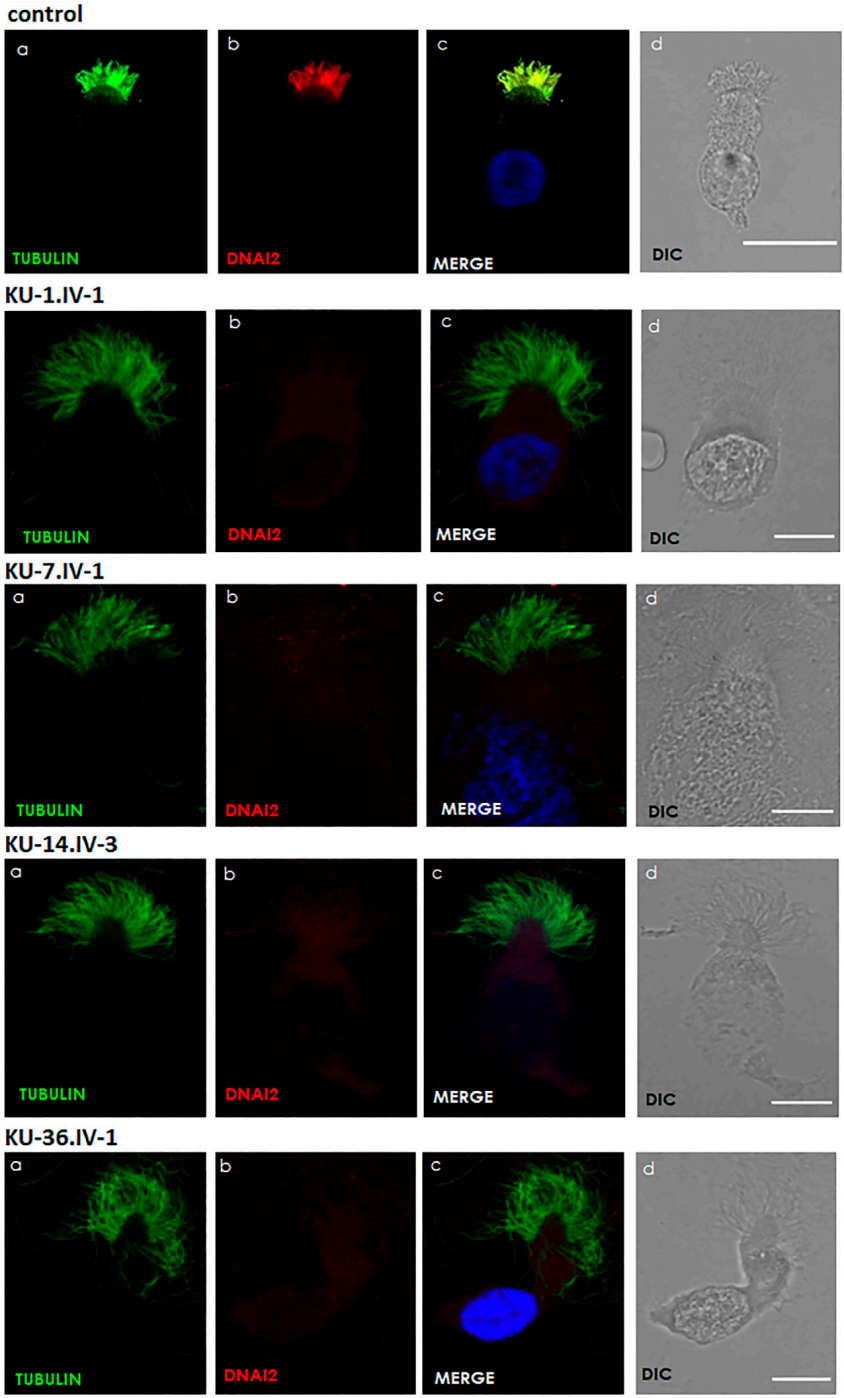
Immunofluorescence images of primary respiratory epithelial cells for PCD individuals. The IF was performed using monoclonal anti-acetylated α tubulin: panel **(A)** and polyclonal anti-DNAI2: panel **(B)** antibody for selected PCD individuals *vs.* healthy controls. As seen in the control, the merged images: panel **(C)** show a yellow co-staining within the ciliary axoneme which indicates that both proteins co-localized within respiratory cilia compared with the images for PCD individuals that demonstrate an absence of anti-DNAI2 staining. Scale bar is 10 µm. **(D)** shows differential interference contrast figures (DIC) for analyzed cells.

**FIGURE 6:**
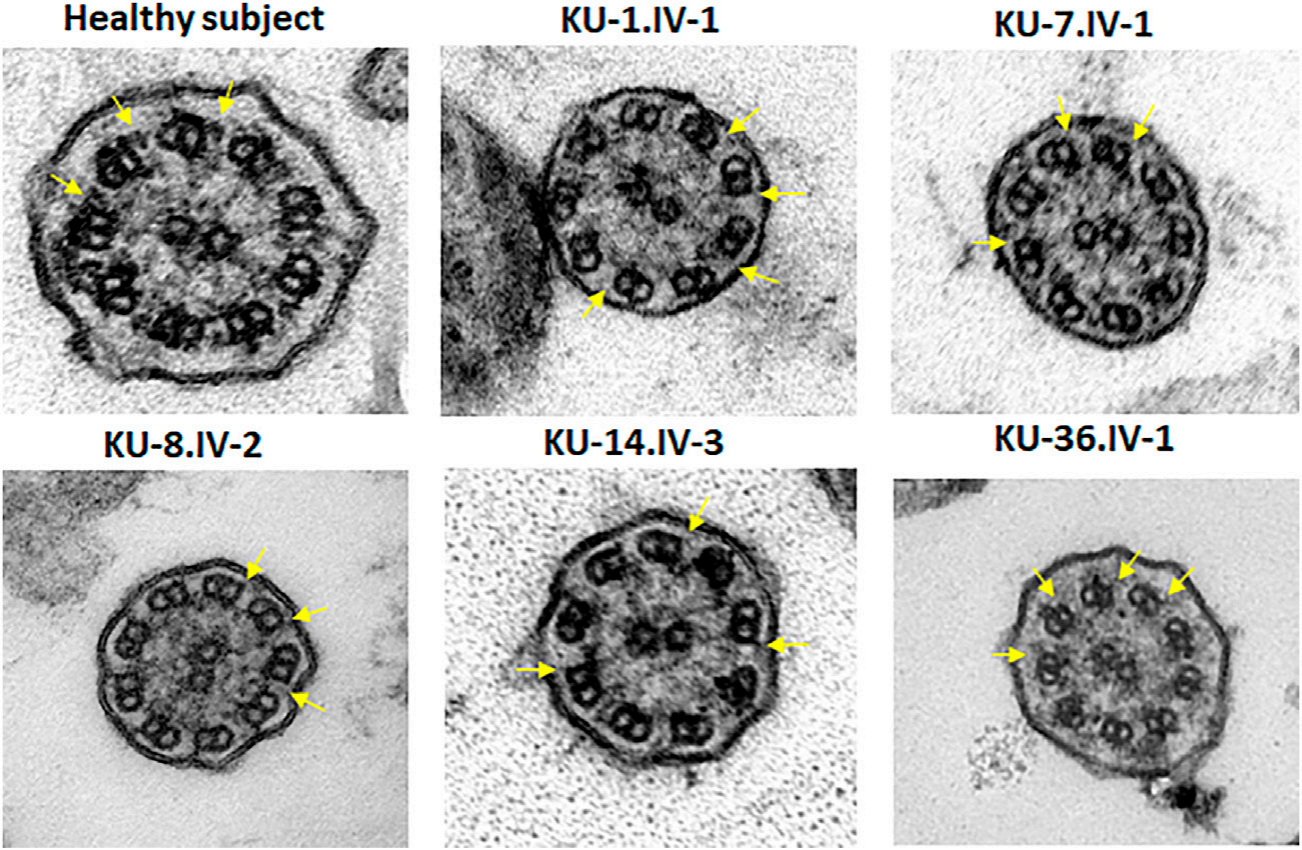
TEM sections of the ciliary axoneme for PCD individuals versus control. TEM results for ciliary axoneme of the ciliated epithelial cells of respiratory system indicate that the PCD patients have an absence of ODAs compared with control, which is mainly seen in patients with defects in genes encoding dynein arm components, consistent with their genotypes.

TEM and IF analyses were performed on nasal biopsies derived from five PCD individuals that carry the homozygous missense variant *DNAI2*:c.740G>A to identify any structural abnormalities of the ciliary axoneme of respiratory epithelia*.* IF results showed negative staining for DNAI2, DNAI1 and DNAH5 proteins in the cilia indicating the absence of outer dynein arm components in all PCD individuals*,* as seen in Figure 5, Supplementary Figures S1, S2. However, results of IF staining showed a normal localization of GAS8 and RSPH9 that are routinely checked in PCD individuals with dynein arm genes defect **(**data not shown). This indicates that these proteins are localized normally in the cilia of the affected individuals, as in control cells. TEM analysis also demonstrated lack of ODAs (Figure 6), consistent with previously published report (Loges et al., 2008).

## Discussion

Autozygosity mapping is a powerful technique for determining the disease-causing founder variants in related patients. Practically, it is more successfully used for investigating the pathogenic variants in distantly related individuals than in closely related individuals, such as affected siblings or cousins, especially in an inbred population, such as the Kuwaiti population. Since patients in multiplex families have multiple IBD intervals, sometimes in multiple chromosomes as frequently seen in our patients, mapping of the disease-gene can be challenging, especially considering the huge volume of sequence variant data generated from next generation sequencing experiments (Williams and Hegde, 2013; Johansen Taber et al., 2014). Autozygosity mapping is also the most efficient strategy for mapping the deleterious founder variants in patients with rare autosomal recessive disorders (Lander and Botstein, 1987; Abecasis et al., 2002; Carr et al., 2011a). Here, we successfully applied AgileMultiIdeogram and AgileVCFMapper software, the latter enabling to visualize small ROH intervals that could not be visualized with other software solutions, to identify a founder variant in *DNAI2* inherited within a single Arabic tribe in Kuwait. This strategy was previously successful in the identification of two novel PCD-loci *CCDC103* and *LRRC6* genes in UK-Pakistani populations (Panizzi et al., 2012; Zariwala et al., 2013) and *CCNO* causing oligocilia with *situs solitus* in a multiplex Kuwaiti family having four PCD individuals (Wallmeier et al., 2014).

Using this strategy we identified novel homozygous missense variant (*DNAI2*:c.740G>A; p.Arg247Gln)) in exon 7 of *DNAI2*. We mapped this *DNAI2* variant in five unrelated consanguineous Arab families, most of them having a family history of the disease. Although this variant is annotated with rs755060592, it is still considered to be a novel variant since the allele frequency of G (normal allele) is 100% in all ethnic backgrounds. All patients harbouring this variant in *DNAI2* (*DNAI2*:c.740G>A) belong to the same tribe, despite samples of these families have been obtained from different hospitals in Kuwait. This suggests that the abovementioned variant in *DNAI2* is a founder hereditary variant running in one tribe that has practiced consanguineous marriages among the members of the tribe over generations and is considered to be particularly isolated, i.e., never married outside the tribe due to historical issues. Immunofluorescence analyses indicates that axonemal assembly of both proximal and distal localized ODA types 1 and 2 does not take place, demonstrated by complete absence of DNAI2, DNAH5, and DNAI1 in ciliary axonemes of affected individuals. TEM analysis on cilia cross-sections confirms lack of ODAs as ultrastructural defect in these individuals.

Previously published data about DNAI2 function characterized DNAI2 as relevant component of ODA type 1 and ODA type 2 in human (Fowkes and Mitchell, 1998): ODAs are the driving force for generation of the ciliary beats. ODAs and IDAs are composed of multimeric protein complexes that are pre-assembled in the cytoplasm prior to transport into and docking onto the axonemes (Fowkes and Mitchell, 1998). The human respiratory cilia have two different ODA heavy chains (HCs): ODA type 1 in the proximal part of the axoneme, composed of γ-HC DNAH5 and β-HC DNAH11, and ODA type 2 in the distal part of the axoneme, composed of β-HC DNAH9 and γ-HC DNAH5 (Loges et al., 2018). IF studies on respiratory cilia show subcellular localization of DNAI2 throughout the entire length of ciliary axonemes, demonstrating that DNAI2 is a component of both ODA type 1 and ODA type 2 complexes. In addition, previous IF analyses of respiratory cilia of PCD individuals with bi-allelic pathogenic nonsense and frameshift *DNAI2* variants showed a complete absence of not only DNAI2, but also of DNAH5, component of both ODA type 1 and ODA type 2 and of DNAH9, component of ODA type 2, demonstrating that DNAI2 is necessary for the correct ODA assembly of both ODA types. In contrast to DNAI2, the Dynein Axonemal Intermediate Chain 1 (DNAI1) is relevant for the assembly of especially ODA type 2 as shown by restriction of both DNAH5 and DNAI2 to the proximal part of the axoneme and lack of DNAH5 and DNAI2 in the distal part of the axoneme in DNAI1 mutant respiratory epithelial cells (Fliegauf et al., 2005; Loges et al., 2008). Thus, DNAI2 is only partially dependent on DNAI1 as it can be assembled in proximal ODAs.

Here, we showed by immunofluorescence analysis complete absence of DNAI2, DNAH5. and DNAI1 in axonemes of affected *DNAI2* individuals identified in this study, demonstrating that this missense variant completely abrogates preassembly of both ODA type 1 and ODA type 2. Electron microscopy analyses confirmed loss of ODAs in these affected individuals. According to ACMG criteria, the *DNAI2* missense variant identified in this study (*DNAI2*:c.740G>A; p.Arg247Gln) has so far been classified as Variant of Uncertain Significance despite high pathogenicity scores. Using these two well-established *in vitro* functional tests, immunofluorescence analysis and electron microscopy, we now provide functional evidence, that this missense variant is pathogenic according to ACMG criteria and cause of PCD in the families presented here.

The identification of this founder variant (*DNAI2*:c.740G>A; p.Arg247Gln) in this Arabic tribe and functional evidence of pathogenicity presented in this manuscript will impact and improve genetic analysis of the Kuwaiti population suspected for PCD, especially from this Arabic tribe.

## Data Availability

The datasets presented in this article are not readily available because ethical, legal and privacy issues prevent deposition in public community supported repositories as they contain data that would allow re-identification of individuals. Requests to access the datasets should be directed to Dalal A. Al-Mutairi (dalal.almutairi@ku.edu.kw).

## Supplementary material

Supplementary Figure S1 Immunofluorescence images of primary respiratory epithelial cells for PCD patients. The IF was performed using monoclonal anti-acetylated α tubulin: panel **(A)** and polyclonal anti- DNAI1: panel **(B)** antibody for selected PCD patients versus healthy controls. As seen in the control, the merged images: panel **(C)** show a yellow co-staining within the ciliary axoneme which indicates that both proteins co-localized within respiratory cilia compared with the images for PCD patients that demonstrate an absence of anti-DNAI2 staining. Scale bar is 10 µm: panel **(D)**.

Supplementary Figure S2 Immunofluorescence images of primary respiratory epithelial cells for PCD patients. The IF was performed using monoclonal anti-acetylated α tubulin: panel **(A)** and polyclonal anti- DNAH5: panel **(B)** antibody for selected PCD patients versus healthy controls. As seen in the control, the merged images: panel **(C)** show a yellow co-staining within the ciliary axoneme which indicates that both proteins co-localized within respiratory cilia compared with the images for PCD patients that demonstrate an absence of anti-DNAI2 staining. Scale bar is 10 µm: panel **(D)**.

Supplementary Figure S3 Allelic discrimination Plot for DNAI2 Mutation. The six patients showed homozygous mutant type pattern (AA; red dots) while their parents showed heterozygous mutant and normal alleles pattern (GA; green dots) consistent with Sanger sequencing data. A control DNA panel composed of 100 DNA samples collected randomly from healthy Arab individuals from different areas in Kuwait were run in the assay and the results showed all the control samples carry the wild type allele in homozygous pattern (GG; blue dots). This indicates that this mutation is very rare and even the carriers are not existing in Arabs.
