# Supplementary material for: Identification of a novel founder variant in DNAI2 cause primary ciliary dyskinesia in five consanguineous families derived from a single tribe descendant of Arabian Peninsula
Source: Front Genet. 2022 Oct 10;13:1017280. doi: 10.3389/fgene.2022.1017280 (PMC9596166; doi:10.3389/fgene.2022.1017280)
Supplement: Supplementary file 1 [file Table1.DOCX]

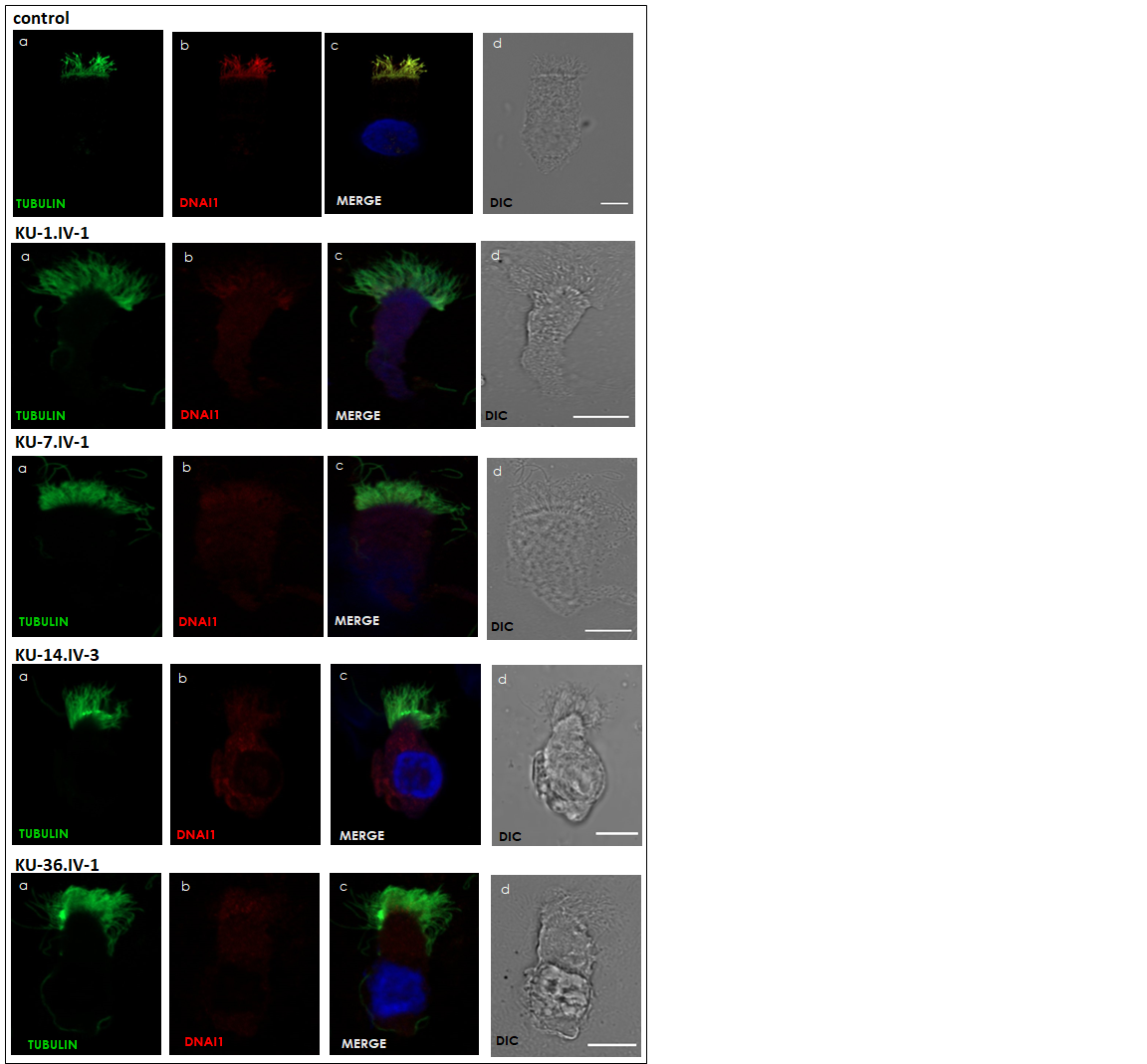


**Figure S1: Immunofluorescence images of primary respiratory epithelial cells for PCD patients.** The IF was performed using monoclonal anti-acetylated α tubulin: panel (a) and polyclonal anti- DNAI1: panel (b) antibody for selected PCD patients versus healthy controls. As seen in the control, the merged images: panel (c) show a yellow co-staining within the ciliary axoneme which indicates that both proteins co-localized within respiratory cilia compared with the images for PCD patients that demonstrate an absence of anti-DNAI2 staining. Scale bar is 10 µm: panel (d).


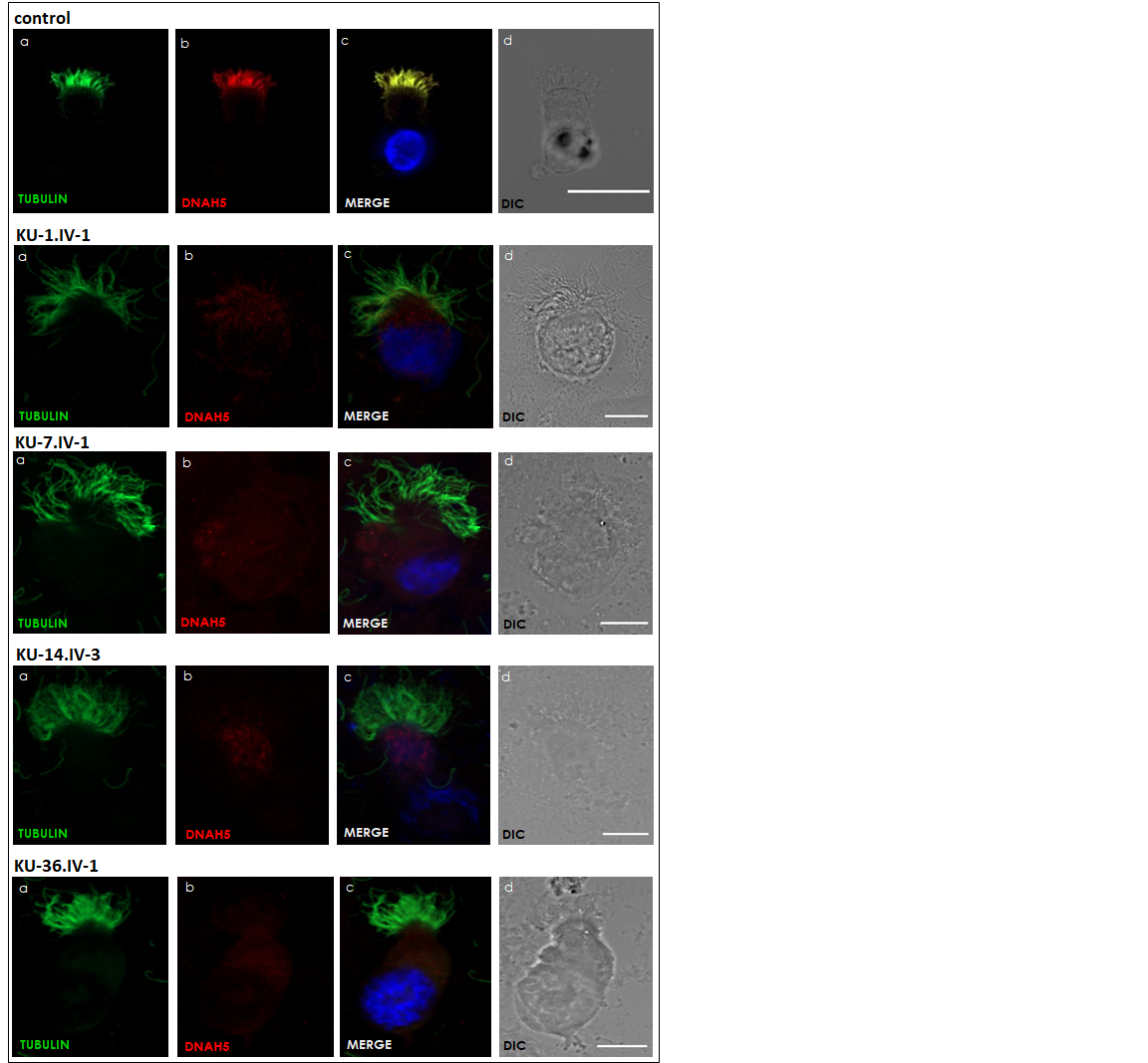


**Figure S2: Immunofluorescence images of primary respiratory epithelial cells for PCD patients.** The IF was performed using monoclonal anti-acetylated α tubulin: panel (a) and polyclonal anti- DNAH5: panel (b) antibody for selected PCD patients versus healthy controls. As seen in the control, the merged images: panel (c) show a yellow co-staining within the ciliary axoneme which indicates that both proteins co-localized within respiratory cilia compared with the images for PCD patients that demonstrate an absence of anti-DNAI2 staining. Scale bar is 10 µm: panel (d).


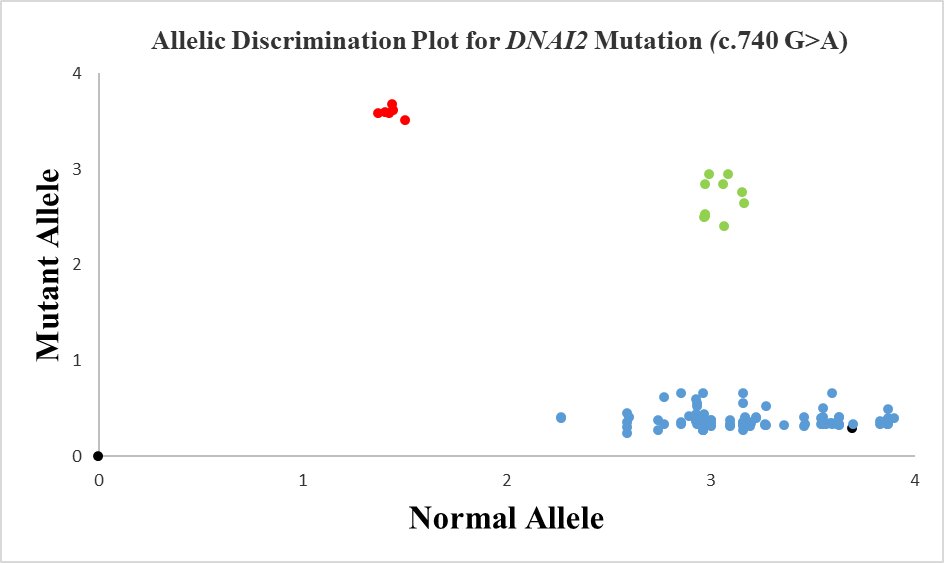


**Figure S3: Allelic discrimination Plot for *DNAI2* Mutation**. The six patients showed homozygous mutant type pattern (AA; red dots) while their parents showed heterozygous mutant and normal alleles pattern (GA; green dots) consistent with Sanger sequencing data. A control DNA panel composed of 100 DNA samples collected randomly from healthy Arab individuals from different areas in Kuwait were run in the assay and the results showed all the control samples carry the wild type allele in homozygous pattern (GG; blue dots). This indicates that this mutation is very rare and even the carriers are not existing in Arabs.
